# Diagnostic Modalities in Critical Care: Point-of-Care Approach

**DOI:** 10.3390/diagnostics11122202

**Published:** 2021-11-25

**Authors:** Sasa Rajsic, Robert Breitkopf, Mirjam Bachler, Benedikt Treml

**Affiliations:** 1 General and Surgical Intensive Care Unit, Department of Anaesthesiology and Critical Care Medicine, Medical University Innsbruck, 6020 Innsbruck, Austria; sasa.rajsic@i-med.ac.at (S.R.); mirjam.bachler@tirol-kliniken.at (M.B.); 2 Transplant Surgical Intensive Care Unit, Department of Anaesthesiology and Critical Care Medicine, Medical University Innsbruck, 6020 Innsbruck, Austria; robert.breitkopf@tirol-kliniken.at

**Keywords:** bedside, critical care, critically Ill, diagnostic modalities, intensive care unit, imaging procedures, laboratory monitoring, point-of-care, POC

## Abstract

The concept of intensive care units (ICU) has existed for almost 70 years, with outstanding development progress in the last decades. Multidisciplinary care of critically ill patients has become an integral part of every modern health care system, ensuing improved care and reduced mortality. Early recognition of severe medical and surgical illnesses, advanced prehospital care and organized immediate care in trauma centres led to a rise of ICU patients. Due to the underlying disease and its need for complex mechanical support for monitoring and treatment, it is often necessary to facilitate bed-side diagnostics. Immediate diagnostics are essential for a successful treatment of life threatening conditions, early recognition of complications and good quality of care. Management of ICU patients is incomprehensible without continuous and sophisticated monitoring, bedside ultrasonography, diverse radiologic diagnostics, blood gas analysis, coagulation and blood management, laboratory and other point-of-care (POC) diagnostic modalities. Moreover, in the time of the severe acute respiratory syndrome coronavirus 2 (SARS-CoV-2) pandemic, particular attention is given to the POC diagnostic techniques due to additional concerns related to the risk of infection transmission, patient and healthcare workers safety and potential adverse events due to patient relocation. This review summarizes the most actual information on possible diagnostic modalities in critical care, with a special focus on the importance of point-of-care approach in the laboratory monitoring and imaging procedures.

## 1. Introduction

The concept of intensive care originates from the disastrous Copenhagen polio epidemic in 1952, when hundreds of patients required mechanical ventilation for several weeks due to the respiratory failure. At this time, artificial ventilation was provided manually by medical and dental students, as the access to respirators was very limited. Due to the acute and immense increase in the number of critically ill patients, Bjorn Ibsen organized the first intensive care unit (ICU) in Europe, gathering together staff of diverse medical specialties to take care of these patients. The implementation of positive pressure ventilation entailed the need for a better monitoring of the patient’s pulmonary gas exchange [1]. Therefore, the arterial blood gas analysis was developed as one of the first point-of-care (POC) diagnostics, by the invention of the Clark- and Severinghaus-electrodes and a pH monitoring technology, which was interestingly developed by the Carlsberg factory in Copenhagen, the representative of Danish brewing industry. Finally, this organized form of critical care medicine and rapid diagnostics drastically reduced the polio mortality [1]. With the time, the concept of ICU was spreading worldwide starting with the first four-bed “shock ward” established in the early 1960s in the United States of America [2] and the United Kingdom.

Since then, critical care has more or less become a race against time. Ranging from emergency medical care to advanced trauma life support and in-hospital rapid response teams, the patients’ outcome is increasingly depending on early diagnostics and immediate medical treatment. By rising the immediate survival rates and continuously providing better ways to support and replace even multiple organ systems, modern intensive care units are not only further increasing their own demand but also healthcare costs by prolonging the patients’ length of stay [3,4,5].

The severity of the illness and its need for immediate clinical decision-making, use of various invasive machine life-support configurations and its associated higher risks for hospital acquired infections and patient safety during intra-hospital transports or isolation measurements for patients with infectious diseases often necessitate diagnostic testing to be done directly bed-side, at the point of patient care.

In the time of the ongoing pandemic, caused by severe acute respiratory syndrome coronavirus 2 (SARS-CoV-2 virus), POC diagnostic techniques became crucial for time-saving evaluation of acute respiratory distress, without overwhelming the already overloaded ICU staff by avoidable in-hospital transports putting both the severely ill patients and the hospital employees on additional risk. Adverse events are common in both out- and intra-hospital transport, most commonly being associated with the equipment malfunctions [6]. Infectious diseases such as coronavirus disease 2019 (COVID-19) entail the risk of further pathogen transmission by exposure of healthcare staff, patients and potential visitors.

In this review, we summarize and discuss the most current information on possible diagnostic modalities in critical care, with a special focus on the importance of point-of-care approach in the laboratory monitoring and imaging procedures, including their advantages and limitations. Furthermore, we emphasize the significance of POC testing and diagnostics in the setting of highly transmittable infectious diseases like COVID-19, where the availability, diagnostic capacity, speed, accuracy and costs imply limiting factors in patient care.

## 2. Point-of-Care Diagnostic

Point-of-care diagnostic techniques are rapidly emerging as important and irreplaceable tools in the hands of intensive care physicians. The POC approach is defined as a medical diagnostic procedure that is performed near or at the site of patient care (bedside) potentially leading to an immediate modification of the ongoing therapy [7,8], outcome improvement and a reduction of morbidity and mortality [9,10,11]. The benefit correlates with the severity of the disease.

### Advantages and Dissadvantages of POC

Every medical technology has its strengths and weaknesses. Depending on the affected group, advantages and disadvantages of bedside diagnostics can be further observed from the patient, healthcare workers and government or healthcare funder perspective [12,13,14,15], see Table 1.

From the patient point of view, the most important advantage is the speed of diagnosis with consequent potential reduction of treatment delay, length of stay, morbidity and mortality. An underestimated patient related advantage is lesser blood loss due to sampling for the laboratory analysis, as the POC diagnostic usually requires smaller sample volume. Intensive care patients may lose up to 340–660 mL of blood per week of intensive care [16,17] due to diagnostic blood sampling, which is associated with an increased probability of blood transfusion [18]. The most of the collected blood sample (91%) is discarded in the diagnostic process [19]. This problem becomes essential in neonatal and paediatric critical care [20]. Another important aspect is patient safety, being especially raised in the SARS-CoV-2 pandemic, as every relocation of patient could result in severe adverse events, and may present additional hazards for the patient’s surroundings. The main disadvantages are the costs (in e.g., out-of-pocket healthcare model) and need for additional diagnostics.

From the healthcare workers perspective, the biggest benefit is the possibility of immediate recognition and treatment of medical conditions, which is essential in the management of life-threatening conditions. On the other hand, a complex training, regular use and recertification are needed for proper results. This leads to additional work load for the caregivers and higher expenditures for the healthcare system. To take advantage of the time saving potential, it is important not to delay the therapy through inexperienced investigators or unregulated processes. This is one of the main reasons why the European Resuscitation Council is still very reluctant to recommend POC use in resuscitation, although its 2021 guidelines already state that the bedside techniques can be used to diagnose treatable causes of cardiac arrest, if used by an experienced operator [21].

**Figure 1:**
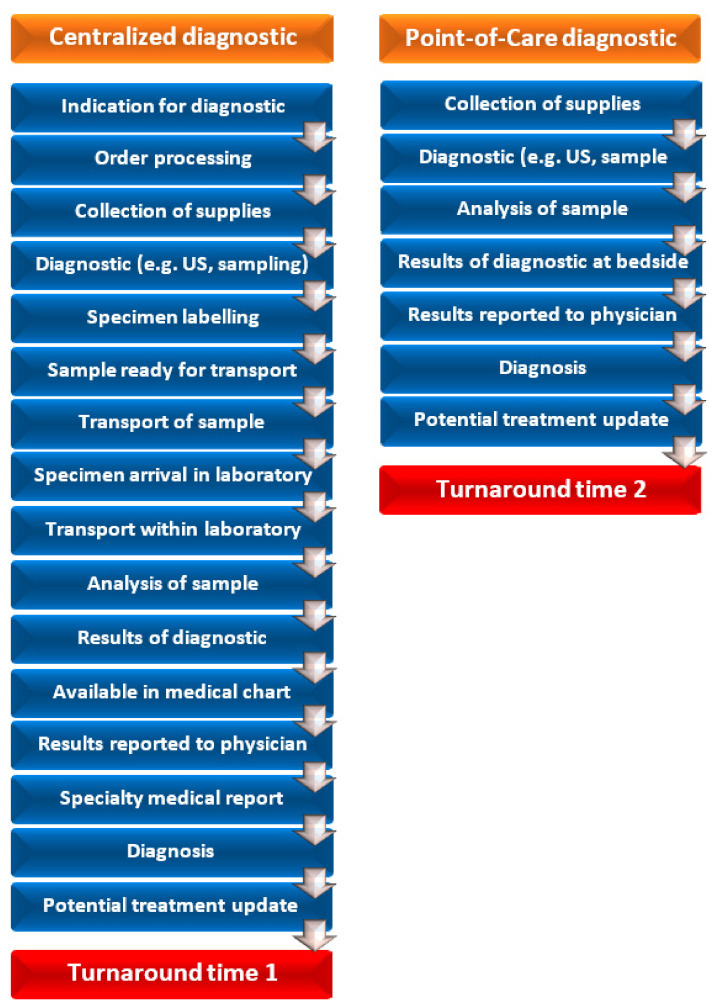
Model of activities comparison of point-of-care and central centralized diagnostics. US reflects ultrasound.

Reduction of turnaround time is another benefit of POC testing. The use of blood gas analysers is a prototype of a simple POC diagnostic drastically reducing turnaround time and resulting in a better control of ventilation, electrolytes and acid-base disorders [22]. Even if continuous pulse oximetry and capnometry could reduce need for blood gas analysis, the discrepancy in results of capnometry (end tidal CO_2_) and blood gas analysis (PaCO_2_) in critically ill patients can be disastrous if unrecognized, especially in neurosurgical patients [22,23,24,25,26,27]. Turnaround time for blood gas analysis was significantly longer if done in a centralised laboratory (27.3 min), in comparison to POC approach (6.8 min). In the case of electrolytes analysis, the turnaround time was 95 min if done in a laboratory, and only 7.1 min in the case of POC (Figure 1) [14].

From the government or healthcare funder viewpoint, the greatest benefit can be seen in the reduction of overall treatment costs besides the expenditures for research, development and acquirement of POC diagnostics.

Nevertheless, the validity of the test results must be guaranteed, especially in the life-threatening field of critical care. Up to 70% of medical decisions are based on laboratory tests [28] and improved patient outcomes with increased speed of healthcare delivery can only be guaranteed if the POC diagnostic is performed with sufficient expertise and the delivered results are accurate and reliable. Otherwise, there is a potential for mismanagement and increased risk of adverse events [29].

Finally, due to fast development of new technologies, diagnostics are increasingly continuing to focus on bedside techniques, bringing the technology from in-hospital not only to pre-hospital care but even to patients’ homes, resulting in even faster recognition of potential life-threatening medical conditions and further reducing treatment delay. This evolution imposes the need for constant medical education, collaboration between different health care providers, information consolidation and share, not only in critical care, emergency medicine, or other acute care settings but also in the patient’s home.

In the following paragraphs, an overview of the laboratory testing and imaging POC diagnostic modalities and approaches in an ICU setting are presented.

## 3. Point-of-Care Diagnostics in Haematology and Biochemistry

The main advantage of POC approach in haematology and biochemistry diagnostic in an ICU setting is an extremely reduced turnaround time, being an essential factor for acute care of patients. POC diagnostics have a potential for prompt, precise, reliable and accurate analysis of critical biomarkers which speeds up decisions and improves patient related outcomes through real-time management of the physiological deterioration.

The most often used POC diagnostic methods in critical care are now briefly described.

### 3.1. Arterial Blood Gas Analysis

One of the major concerns in the care of the critically ill is the maintenance of tissue oxygenation and preservation of a normal acid-base status. Acute changes in these parameters (partial pressure of oxygen-pO_2_, partial pressure of carbon dioxide-pCO_2_ and pH), can lead to serious tissue injuries and death if not recognized and treated [30,31]. Therefore, rapidly available results may be crucial for effective monitoring and treatment of patients under risk. Numerous studies investigated benefits of arterial blood gas POC analysis, and a prospective study from USA reported on a 50% reduction of mortality when POC is used (compared to central laboratory) after congenital heart surgery [32]. Few studies showed improved patient outcome and marked mortality reduction with the use of early goal-directed therapy in septic patients. This was based upon POC determination of lactate levels, central venous oxygen saturation and pH [33,34]. Finally, the Laboratory Medicine Practice Guidelines of the National Academy of Clinical Biochemistry recommends the use of POC blood gas analysis as a way to improve outcomes in ICU patients [35].

The arterial blood gas can also be continuously monitored, which is useful during surgical procedures with rapid changing blood gases (during one lung ventilation, cardiac surgery or organ transplantation). In the case of the critically ill, continuous blood gas monitoring could be beneficial in cases where frequent monitoring is needed but limited with iatrogenic blood lost (neonatal and paediatric ICU, acute respiratory distress syndrome, etc.). Despite several advantages of this system, high cost of monitoring is a major factor limiting the widespread use [36].

As correct interpretation of arterial blood gas analysis needs profound knowledge and experience, new digital solutions may be helpful in the daily clinical routine. The arterial blood gas algorithm (ABG-a) presents a real-time interpretation software of oxygenation, acid-base status, and renal function. This technological feature can make understanding and interpretation of blood gases faster and easier. Use of ABG-a could help healthcare professionals to improve workflow and even patient safety by reducing potential human errors at overwhelmed ICUs with limited personal and equipment resources [37].

### 3.2. Co-Oximetry and Haematology

Blood gas analysers and co-oximeters are commonly integrated into one device. The co-oximeter is able to measure haemoglobin content and values related to the haemoglobin binding in the blood sample (percentage of oxygenated and deoxygenated haemoglobin, carboxyhaemoglobin and methaemoglobin). Using the blood oxygen saturation (SaO_2_) and the mixed venous oxygen saturation (SvO_2_), the total arterial oxygen content and arteriovenous oxygen difference can be calculated (the amount of oxygen consumed by tissues). Earlier sepsis guidelines suggested monitoring of SvO_2_ and central venous oxygen saturation (ScvO_2_) and maintaining ScvO_2_ above 70% [38] and SvO_2_ ≥65% in septic patients [39]. However, this recommendation needs to be taken with caution as high levels of ScvO_2_ may reflect an inadequate use of oxygen and is associated with increased mortality in the latter stages of septic shock [40].

Methaemoglobin levels may be a useful marker in the fast diagnosis of sepsis or septic shock onset [41]. Furthermore, elevated methaemoglobin levels may also occur during use of some local anaesthetics (prilocaine, benzocaine, tetracaine and lidocaine) [42] and can cause hypoxic encephalopathy, myocardial infarction or even death.

Another application of co-oximetry is the detection of carbon monoxide intoxication, as the pulse oximetry overestimates the oxygen saturation [43].

Co-oximetry is used regularly as POC haemoglobin and haematocrit testing, which is important for the prompt accomplishment of transfusion requirements according to modern patient blood management practice. As anticipated benefits of blood transfusion must be weighed against potential transfusion-related complications, an accurate and prompt measurement of haematocrit and haemoglobin is crucial for clinicians’ decision making.

Most of the POC devices require periodical blood sampling, but some are suitable for continuous haemoglobin assessment (based on co-oximetry/spectrophotometry). The gold standard of haematocrit measurement is centrifugation, used mostly in centralized laboratory, where the red cells are separated from the plasma by centrifugal force. Another technique is to calculate the haematocrit after measuring the average size and number of the red blood cells by performing a complete blood cell count based upon the Coulter impedance principle, where the single layer of red blood cells passes through a pair of electrodes that measure electrical resistance and consequently haematocrit. However, the conductivity and co-oximetry are the main POC methods used for haematocrit and haemoglobin determination. In a conductometric method, the electrical conductivity is inversely related to the haematocrit, and haemoglobin is calculated indirectly, as a function of haematocrit. The main disadvantage of this method is impaired accuracy by the use of heparin, crystalloid or colloid haemodilution, leucocytosis and altered sodium or protein levels, all conditions regularly seen during and after complex surgeries (organ transplantation, cardiac surgery, trauma management with massive transfusion, etc.) and in critically ill patients [44,45,46]. Co-oximetry analysers use the multiple wavelength spectrophotometry and measure haemoglobin level, from which haematocrit value is calculated. Spectrophotometry may scarcely be affected by high lipids and cellular fragments from incomplete haemolysis. The measurement of haemoglobin using co-oximetry is in literature described as the most accurate, consistent and reliable method in special groups of intensive care and surgical patients [45,47].

As the use of different POC technology can lead to false low or (in worst case) false high haematocrit and haemoglobin values, clinicians should be aware of the type of POC device and method used, and likewise be cautious in relying solely on the POC data when making decisions on blood transfusion.

Further POC diagnostics for complete blood count are outside of this review scope.

### 3.3. Electrolytes

Disturbances in electrolyte homeostasis are common in the critically ill, and can precipitate life-threatening events if not timely recognized. Electrolytes measured on a regular basis at ICU include sodium, potassium, chloride, calcium, magnesium, hydrogen phosphate and hydrogen carbonate.

Changes in sodium, potassium and chloride are the electrolyte disorders most often diagnosed in the critically ill, with hyponatremia being the most common one. Almost one third of patients are affected, the clinical presentation may include a broad spectrum of symptoms, from mild cognitive deficiencies, over encephalopathy and central pontine myelinolysis to brain herniation [48]. The appropriate monitoring and management of dysnatremia should be provided through its timely diagnosis, a careful analysis of the underlying causal mechanisms, and the recognition of its severity with potential of a negative impact on the patient’s course.

Central laboratories rely classically upon indirect ion selective electrode analysers. This involves diluting the blood sample and the assumption that the aqueous phase of blood represented 93%. Under certain circumstances, the remaining 7% of dissolved solids, mostly proteins and lipids, may raise. With such hyperproteinaemia and hyperlipidaemia, the volume of plasma solids increases leading to falsely low values for serum electrolytes, especially sodium. Such pseudohyponatremia is reliably detected applying direct ion potentiometry.

Potassium is perhaps the most frequently supplemented electrolyte, being essential for normal functioning of the cardiovascular and nervous system, skeletal muscle and internal organs in general. Potassium concentration gradient, intracellular 140 and in serum 3.5–5.5 mmol/L, present a main determinant of the resting membrane potential across cell membranes. In critically ill care, maintenance of potassium homeostasis can lead to reduction of cardiac arrhythmias and reduced ICU mortality [49]. The vast majority of patients with potassium disorders are asymptomatic, but the clinical presentation may range from neuromuscular weakness to lethal arrhythmias and cardiac arrest [50]. Furthermore, despite vigorous quality control processes, preanalytical errors can lead to false elevation of potassium in reported results, with haemolysis as one of possible causes of this pseudohyperkalemia. Pseudohyperkalemia can be identified in the central laboratory, but not with POC testing [51]. Moreover, the difference between whole blood electrolytes measured by POC devices and serum electrolytes measured in the central laboratory is described as statistically but not clinically significant [34]. The benefits of fast provided results in an emergency setting overcome the potential difference in accuracy. The impeccable example for this advantage is identification of life-threatening electrolyte disorders as a reversible cause of cardiac arrest in a reanimation situation (hyper-/hypokalaemia, and seldom hyper-/hypocalcaemia and hyper-/ hypomagnesaemia). Moreover, the ERC Guidelines 2021 recommend an immediate check for hyperkalaemia using POC testing in a life-threatening situation [21]. Finally, chloride, the forgotten electrolyte, receives a limited amount of attention in comparison to other routinely measured electrolytes, even though hypochloraemia is a common finding and most often caused by infusion of chloride-rich fluids [52].

The POC monitoring of ionized calcium is extraordinarily important for the management of citrate anticoagulation to prevent clotting in the extracorporeal circuit during continuous renal replacement therapy, during transplantation, cardiac or other surgical procedures [35]. In primary resuscitation of trauma patients, treatment of hypocalcaemia presents part of preventing “death triad”, where each triad factor has a place in a vicious cycle, exposing patients to a high risk of death [53].

Magnesium has an important role in several biochemical and physiological processes, including conduction of electrical potential and muscular cell contraction. Hypomagnesaemia is a common finding in critically ill patients, associated with increased mortality and prolonged hospitalization [54]. In an ICU setting, patients with any type of cardiac illness, rhythm disorder, asthma, vasospasm, cramps, seizures, preeclampsia/eclampsia, stroke, digitalis toxicity, etc. can benefit from close magnesium monitoring and substitution. In case of arrhythmias, prompt determination of magnesium levels can lead to causal treatment of life-threatening heart rhythm disorders and improve outcome [35].

An important superiority of POC electrolytes analysis is the smaller blood sample [35], especially important in neonatal and paediatric populations of the critically ill, where it can lead to reduction of iatrogenic blood loss and transfusion rates. However, intensivist should be aware of the potential difference in the POC and central laboratory measured electrolytes, with rather negligible consequences in decision-making.

### 3.4. Lactate

Lactate is an essential metabolite of glycolysis and oxidative phosphorylation, the main energy producing processes. The relationship between stress, oxygen delivery, tissue hypoxia and increase of lactate is well described in the literature [55,56], making this parameter one of the most important in estimation of disease severity, morbidity and mortality prediction and finally monitoring of treatment adequacy. In an ICU setting, a poor lactate clearance may indicate reduction of systemic oxygen delivery. In sepsis, the Surviving Sepsis Campaign: International Guidelines for Management of Sepsis and Septic Shock 2021 recommended lactate guided resuscitation, with the goal of lactate normalisation in patients with elevated lactate levels [38]. The use of lactate monitoring in a goal-directed therapy can improve clinical outcome and significantly reduce mortality, making it a valuable POC parameter in the early resuscitation of the critically ill [55,57].

### 3.5. Glucose

Regulation of blood glucose is a subject of discussion since more than two decades, where earlier standard of care was use of intensive insulin treatment targeting normoglycaemia (80–110 mg/dL) [58], followed with the moderate glucose control strategies (180 mg/dL or less) [59], both with mortality reduction as a goal. Current guidelines recommend initiation of continuous insulin therapy for blood glucose (BG) levels of ≥180 mg/dL and targeted to a BG range of 100–150 mg/dL [60]. Moreover, insulin induced glycaemic variability should be minimized and potential hypoglycaemia promptly detected and avoided. These recommendations are based on an expert consensus, as the quality of available evidence in the literature is very low.

Regular BG monitoring has its special place in the care of ICU patients. The most recent guidelines recommend BG monitoring every one to two hours for patients receiving continuous insulin infusion. In the case of stabile ICU patients, with BG levels within the desired range and constant insulin infusion rate, the frequency of monitoring can be reduced to every four hours. However, every delay in BG measuring may contribute to the risk of severe hypoglycaemia, and increased mortality [60]. Iatrogenic blood loss and personnel time required for monitoring are the main disadvantages of more frequent testing.

Blood glucose can be measured with diverse POC glucose meters, from capillary BG analysis, or integrated in the blood gas analysis to continuous inline glucose measurement [61]. Accuracy of POC glucose meters compared with the central laboratory showed significant variability in BG levels. The acceptable error according to the International Organisation for Standardisation guidelines is quite high, up to 15 mg/dL variance (for BG under 75 mg/dL) and up to 20% (when BG higher than 75 mg/dL) of the central laboratory analyser [62]. Additional factors at ICU can lead to error in measurement, as, for example, PaO_2_ higher than 100 mmHg can falsely lower readings on the POC [63], low haematocrit can lead to an overestimation of BG level [64], and different medication and metabolic products can further interfere with the accuracy of some POC BG meters [65].

The arterial whole blood sampling is a recommended alternative for finger-stick capillary blood testing for critically ill who are in shock, hypotension, with severe peripheral oedema, vasopressor therapy or any patient with continuous insulin infusion. The finger-stick testing as an invasive procedure is associated with pain, and should be avoided or used as a last option, where no better alternative is available [60].

Continuous glucose sensors in the critically ill provide constant monitoring of BG levels, providing a basis to prevent severe hypoglycaemia and hyperglycaemia, which can further lead to reduced glycaemic variability, reduction in blood sampling, improved outcome and reduced workload for personal and can be cost effective [66,67,68] without major safety concerns [67,69]. During the COVID-19 pandemic, continuous BG monitoring was described as feasible with acceptable accuracy to identify trends and guide insulin therapy in an ICU [70]. An important advantage of this POC approach in the COVID-19 setting is reduction of infection transmission risk for healthcare workers and saving of personal protective equipment, being paramount in the period of global shortage. Continuous BG monitoring can be considered as safe and effective aid in BG management of the critically ill, enabling more rapid and accurate insulin infusion adjustment [66].

In the emergency medicine, POC BG testing can be of enormous importance, where the cause of coma can be immediately diagnosed and treated by the emergency medical team on field [71].

### 3.6. Coagulation

Point-of-care assays are available for a variety of coagulation tests, being generally simple to perform and with rapid turnaround time, which is crucial in acute and critical care setting. POC guided and factor-based coagulation management is a gold standard in praxis, and bedside methods can be divided into viscoelastic, platelet function monitoring and analysis of plasmatic coagulation.

#### 3.6.1. Viscoelastic Methods

Using viscoelastic methods (i.e., rotational thrombelastic system-ROTEM^®^, thrombelastographic system-TEG^®^, ClotPro^®^, Sonoclot^®^ etc.), clinicians are able to detect life threatening coagulopathy manifested with hypofibrinogenaemia, irregularities in coagulation initiation, clot formation and firmness, increased or impaired fibrinolytic activity and platelet level in the whole blood, with the first results within five to ten minutes. All first-generation models required manual pipetting of both blood samples and reagents, being time-consuming and with the potential of errors. With the advance in technology, ready-to-use cartridges are available being easy to use and time saving.

In the case of coagulopathy associated with bleeding, the viscoelastic methods improve goal-directed substitution of blood and coagulation products and reduce mortality and degree of blood products substitution in various diseases [72,73]. In comparison to standard coagulation tests, viscoelastic guided treatment resulted in clear cost-saving and more effective treatment for instance in patients undergoing cardiac surgery and trauma patients [74].

They provide more accurate and faster assessment of coagulation, being at the same time more reliable than standard coagulation tests [75]. Nowadays, the care of certain groups like traumatized or postsurgical patients is inconceivable without POC coagulation diagnostic. However, viscoelastic tests are unable to detect single coagulation factor deficiency except from hypofibrinogenemia [76]. Newer assays (ECA-and RVV-test) available for the ClotPro^®^ device can even discriminate in a dose dependent manner between direct FXa- and thrombin inhibitors in e.g., an emergency room setting [77,78]. In addition, in the setting of Intensive Care Units (ICU), viscoelastic tests provide some advantages not only in the management of bleeding situations. Contrary to the standard coagulation tests, only viscoelastic testing can detect hypercoagulability and hypercoagulability can be identified after trauma and surgery [79] and in infectious diseases such as sepsis or COVID-19 [80,81,82]. Furthermore, with viscoelastic tests, an impaired fibrinolysis (ClotPro^®^ TPA-test) resulting in hypofibrinolysis as measured with maximum lysis or lysis index in the classical extrinsic and intrinsic initiated assays in the different devices can be detected [83,84,85]

Since the hypercoagulability and impaired fibrinolysis is associated with increased risk of thrombosis [79,80,83,84,85], viscoelastic testing is therefore able to identify patients with considerable susceptibility to thrombosis. These patients might benefit from intensified anticoagulation prophylaxis or even from a switch to a direct thrombin inhibitor (off-label use), which might be able to improve the fibrinolytic impairment [86,87].

Nevertheless, special training for use of some models is needed and recommended [72].

Several new technologies are in the development stage for coagulation POC testing, including fluorescent microscopy, microfluidics, photoacoustic detection, electromechanical sensing and micro/nanoelectromechanical systems [88]. The new technological trends should focus on an evolution of a highly accurate, robust, rapid and cost-effective coagulation POC assays, being even more user-friendly and accessible.

#### 3.6.2. Platelets Function Monitoring

Platelet activation, adhesion and aggregation are crucial in primary haemostasis, but also in pathophysiology of vascular diseases. Excessive antiplatelet drug effects can lead to deviations of haemostasis and consequent haemorrhage, while inadequate platelet inhibition can result in vascular occlusion and fatal organ infarction. Finding the balance between a beneficial grade of antiplatelet effect and adequate haemostasis is still challenging. The gold standard of platelet function testing is light transmission aggregometry [89], a complex and slow laboratory method, which requires a relatively large amount of blood volume. Especially paediatric patients and neonates, where this assay might be used to diagnose classic inherited platelet function disorders, are exposed to repeated blood sampling and the resulting blood loss might harm these patients [90].

The available POC tests still have important limitations, and they have only subordinate roles according to the current European guidelines on management of major bleeding and coagulopathy following trauma [91]. However, the POC diagnostics may be of value, as addition to the standard laboratory, in the detection of drugs induced platelet inhibition in all cases where no information in regard to previous antiplatelet agents’ intake is available.

There are different devices for platelets function monitoring, employing diverse technologies. For example, the Multiplate^®^ Analyzer (Roche, Basel, Switzerland) uses multiple electrode aggregometry for the platelet aggregation and inhibition measurement. Several assays are available for diverse drugs with different mechanisms of platelet inhibition, and the generation of results takes from six to ten minutes. The ROTEM^®^ platelet module (Instrumentation Laboratory, Bedford, MA, USA) and TEG^®^ (Haemonetics Corporation, Boston, MA, USA) can be used for detection of cyclooxygenase (COX) inhibitors, adenosine diphosphate (ADP) receptor inhibitors and GpIIb-IIIa antagonists [92]. The Platelet Function Analyzer-100 (PFA-100^®^, Siemens, Munich, Germany) is completely automatized and user friendly, based on platelets adhesion under high shear force [93]. Certain limitations should be familiar to clinicians (i.e., thrombocytopathies, low platelet count and haematocrit) as they can lead to prolonged closure time (CT-the time platelets need to occlude the orifice and block the whole blood flow) and be falsely interpreted as platelets dysfunction [93]. It is important to keep in mind that these devices have limited ability to measure platelet’s hyperreactivity, which might be interesting in regard to prothrombotic diseases [94].

The VerifyNow^®^ platelet reactivity assay (ITC, Edison NJ, USA) is a further fully automated POC test using whole blood samples for monitoring of antiplatelet therapy, providing results also within a few minutes [92], though here the haematocrit might also falsify the test results [95]. Moreover, transport of samples using pneumatic tube transport may affect platelet function testing [96].

The platelet function assessment with POC tests is still unsatisfactory [92]. Further improvement of technology could lead to more accurate, reproducible, affordable and reliable results. This could reduce transfusion of blood products leading to increased safety of patients and positive impact on outcome in certain patient populations.

#### 3.6.3. Plasmatic Coagulation Analysis

Within the methods for plasmatic coagulation testing, only global coagulation assays will be addressed in this review: the activated clotting time (ACT), the prothrombin time (PT) and the activated partial thromboplastin time (aPTT).

The PT and aPTT POC tests have been primarily developed for outpatient monitoring of anticoagulation (warfarin) and continuous heparin therapy. Multiple studies showed that the turnaround time is significantly reduced when POC tests were used, leading to improved patient care [97,98]. Furthermore, the use of whole blood PT and aPTT significantly reduced the incidence of postoperative bleeding, transfusion of blood products and operative time [99]. They are useful in critical care, nonsurgical and emergency setting and provide reliable results [100]. However, high variability in POC PT and laboratory PT after protamine reversal in cardiac surgery, with clinically relevant discrepancy and underestimation of coagulopathy, is reported. Meesters et al. recommended not to use POC diagnostics in the first ten minutes after protamine administration in cardiac surgery [101].

The ACT remains the method of choice for heparinization POC monitoring during cardiac surgery, extracorporeal life support, dialysis, cardiac catheterization laboratory and vascular surgery, as the POC and non-POC PT and aPTT are immeasurable when high concentrations of heparin are present. ACTs use the whole blood sample, have short turnaround time and high clotting time repeatability. ACT is usually measured before starting cardiopulmonary bypass and then repeatedly measured to guide heparin dosing, but also to guide protamine reversal at the end of surgery [102].

The main disadvantage is poor correlation with anti-Xa measures of heparin activity, or heparin concentration in general [103] and, especially during haemodilution or hypothermia, both present during cardiopulmonary bypass. During ECMO, the anti-Xa guided heparin dosing resulted in less circuit clotting and resulted in a significant reduction of costs [104]. Thrombocytopenia, anaemia, presence of platelet inhibitors and membrane receptor antagonists, low antithrombin levels, severe hypofibrinogenaemia, and low temperature can also influence the accuracy of ACT POC diagnostics [105].

#### 3.6.4. Emergency

Finally, certain groups of patients are on long-term anticoagulant therapy, traditionally with vitamin K antagonists (i.e., warfarin) and since 2010 increasingly with direct oral anticoagulants (i.e., apixaban, dabigatran, rivaroxaban, edoxaban) [106]. As the portion of patients with indication for direct oral anticoagulants is constantly rising [107], at one point, some of these patients will require urgent surgery from other reasons. Usefulness of time based guidelines is limited, and there is no specific POC diagnostic for direct oral anticoagulants. However, using non-specific POC diagnostic patients under anticoagulation can be promptly identified in an emergency, without losing time on referent laboratory testing [108]. For example, Coagucheck^®^ (Roche, Basel, Switzerland) can be used to identify relevant plasma concentration (more or less than 30 ng/mL) of rivaroxaban [109], and Hemochron^®^ Signature (ITC, Edison, NJ, USA) has an additional capability for both rivaroxaban and dabigatran (more or less than 50 ng/mL) [108,110].

There is no available POC testing for apixaban [108], and non-specific POC tests are not reliable [111]. Clinically relevant concentrations of edoxaban can be safely excluded by Coagucheck^®^ [112].

In case of vitamin K antagonist based anticoagulation, the prothrombin time is the established method used for monitoring, a technique developed by Quick in 1935 [113]. In order to standardize PT reporting, the international normalized ratio (INR) was introduced by the World Health Organisation (WHO), a concept to calibrate each commercial thromboplastin against a reference [114]. The INR POC testing showed its great value in accelerating initiation of emergency thrombolysis in patients with acute ischemic stroke who are using oral anticoagulants, or where previous anticoagulation status is not available [115].

In conclusion, POC diagnostic of coagulopathy is crucial for coagulation management in acute setting, leading to goal directed therapy and reduction of blood components transfusion and reduced ICU length of stay including mortality and cost saving [92,116].

### 3.7. Cardiac Markers

According to the Task Force for the Universal Definition of Myocardial Infarction (by European Society of Cardiology, American College of Cardiology, American Heart Association and World Heart Federation), the clinical definition of myocardial infarction includes the presence of acute myocardial injury detected by pathological cardiac markers being released in the blood, with the evidence of acute myocardial ischemia [117]. The main cardiac markers include cardiac troponin I (cTnI) and T (cTnT), creatine kinase (CK; creatine kinase myocardial band-CK-MB), myoglobin, lactate dehydrogenase and others. Nowadays, the cardiac troponin is the preferred biomarker for diagnosis of myocardial infarction [118].

Use of rapid qualitative and quantitative tests for identification of cardiac biomarkers in acute coronary syndrome present an alternative to standard laboratory, as it may be performed already on field trough emergency services, in the primary care or in the emergency department and especially in remote setting (i.e., cruise ships), offering unique advantages [119]. Moreover, the National Academy of Clinical Biochemistry recommends implementation of POC cardiac biomarker testing if analysis of cardiac biomarkers is not available in less than one hour [120]. This induced a huge commercial interest in novel POC assays, resulting in numerous POC tests from different manufactures. The combination of POC myoglobin, troponin and CK-MB assays measured at baseline, and at 90 min after the first sample, has been widely evaluated and applied [121], leading to reduction of admission and length of stay in emergency department, increasing successful hospital discharge. The leading symptom—chest pain—is responsible for nearly one quarter of all emergency hospital admissions [122]!

The main advantage of POC assays for cardiac biomarkers is reduction of delay caused by specimen transport in a central laboratory and avoiding the lack of immediate central laboratory availability [123]. The savings in turnaround time, from sampling to receiving results, can range from 47–54 min [124,125]. In comparison to earlier assays, newer technology shows increased sensitivity of tests for cardiac biomarkers due to larger sample volume, prolonged incubation time, use of chimeric antibodies and use of more than two antibodies for detection of the target proteins [126]. This improvement in analytical sensitivity leads to earlier detection of acute myocardial infarction [127] and prompt use of appropriate therapy [120]. However, additional clinical trials are needed to confirm that the rapid provision of test results translates directly into clinical benefits and improved workflow [128]. So far, only a few studies investigated the benefits of POC versus central laboratory testing, showing rather inconsistent results [129,130,131,132,133,134,135,136].

The main limitation of troponin use as a biomarker is the relative late blood level increase after the onset of ischemia. Consequently, patients presenting earlier in the course of myocardial injury may have still undetectable levels of troponin in blood, being at risk of missing the acute coronary syndrome diagnosis and therapy. Therefore, diagnosis of acute coronary syndrome requires serial blood sampling to show the trend of biomarker changes, leading to hospital admission of many patients until the diagnosis is established and resulting in additional health service costs and inconvenience for patients [121]. The National Institute for Health and Care Excellence (NICE) recommends troponin I and T testing on initial patient presentation to hospital and again ten to twelve hours after the onset of symptoms (except in the case of tests for high-sensitivity troponin) [137].

Further research and development in diagnostics of acute coronary syndrome focuses on providing POC tests to identify biomarkers of inflammation, plaque instability or rupture and ischemia, independently contributing to the risk stratification of patients with acute cardiac syndrome. Reports on experimental use of electrochemical paper-based analytical devices for POC detection of cardiovascular disease markers are already available, with high sensitivity, rapid analysis time, portability, and low cost [138].

Finally, rapid cardiac marker assays have fewer benefits in an ICU setting when compared to the prehospital patient care and emergency departments. Critically ill patients are most often not able to claim on chest pain or other symptoms, but are constantly monitored and POC echocardiography can be promptly employed in case of acute cardiac instability.

### 3.8. Acute Infections

Acute and complicated infections are often seen in an ICU setting, and prompt diagnostic and therapy are of immense importance. As etiological biomarkers are generally unreliable, broad-spectrum antibiotics are often administrated empirically until the results of standardized diagnostic are available. Sepsis is defined as a life-threatening organ dysfunction caused by dysregulated host response to infection, responsible for nearly six million deaths worldwide, most of them being preventable [139,140]. International Guidelines for Management of Sepsis and Septic Shock from the Surviving Sepsis Campaign recommend administration of intravenous antibiotics as soon as possible and within one hour in case of sepsis or septic shock, not reporting on POC diagnostic possibilities [38]. With every hour of delay of sepsis treatment, the risk of death increases by almost 8% [141]. State of the art for identification of the infection source in blood consists of blood cultures, usually performed in a central laboratory and with results available in a few days. The available diagnostics include tests for bacterial contamination (bacterial culture, most time consuming), followed by pathogen identification and finally an antibiotic susceptibility test. A faster alternative is detection of pathogen DNA directly from blood sample, still not being a POC device but providing results within 30 to 80 min (Septifast, Roche; DiagCORE and T2 Candida, STAT Diagnostics).

Rather than direct identification of potential pathogen causing infection, plasma circulating proteins (C-reactive protein- CRP, procalcitonin- PCT and interleukins) are used as biomarkers of infection and employed for antibiotic guidance, including lactate level as a marker of altered tissue perfusion. POC devices for CRP and PCT are widely available, with variability in the precision [142]. There are so far no definitive and reliable markers for sepsis identification. POC technologies for direct identification of pathogens exist, but evidence for their impact on outcomes is still not available [143].

Moreover, measurements of lactate levels in cerebrospinal fluid can be helpful in the detection of central nervous system infections, especially in neurosurgical patients. POC blood gas analysers can reliably measure lactate levels in cerebrospinal fluid and lead to timely identification of developing meningitis [144].

Further research should urgently focus on development of POC tests for etiological identification of infections and assays measuring immune response of patient to infection. Here the POC viscoelastic testing might be helpful in the future since impaired fibrinolysis or even fibrinolytic shutdown can discriminate between systemic inflammatory response syndrome (SIRS) and sepsis in critically ill patients [145,146]. Further technologies for new markers of immune response are in the development phase (neutrophil CD64 expression, microfluidic devices, cell motility, microRNA, cell stiffness, etc.), and additional research is needed to evaluate their role in sepsis [143].

More comprehensive reviews focusing especially on sepsis biomarkers, including POC diagnostic, devices for the direct identification and removal of pathogens and potential future development can be found elsewhere [142,143,147].

## 4. Point-of-Care Imaging Procedures

The most often used imaging POC diagnostics at the ICU are ultrasound (US) and portable chest radiography (pCXR). Cardiac and pulmonary pathologies, examination of abdominal organs, identification and evaluation of pleural and pericardial effusions, free fluids and air, pneumothorax, location of indwelling medical devices (endotracheal tube, central catheters, drainages, implantable devices) or any acute instability are clear and usual indications for pCXR or US examination of the critically ill.

The number of studies recommending restrictive application of a routine daily pCXR rule is increasing, as the evidence of impairing outcome, quality of care or patient safety is missing. The American College of Radiology Appropriateness Criteria Expert Panel recommended that the stable ICU patients, including those being mechanically ventilated, should have a pCXR only if there is a clinical indication as they are of low diagnostic contribution, have negligible impact on management decisions, and unexpected relevant findings are scarce [148,149,150,151,152,153]. However, the need for daily pCXR is still the subject of discussion and will remain until the further prospective evidence is available [154]. Nowadays, US is gaining popularity in ICU diagnostics as it decreases the radiation exposure of patients and healthcare workers, is readily available and delivers immediate results especially during imaging controlled interventional procedures. An additional limitation of the pCXR is not only the need for acquisition by trained personnel but also in its immediate interpretation by a specialist trained to evaluate chest radiographs.

Easier access to portable devices combined with the extensive training of physicians leads to a revolutionary increase of its bed-side use on ICUs [155,156]. A term “critical care ultrasonography” has been introduced and defined as a bedside diagnostic or guiding procedure performed and interpreted by the intensivist. It consists of general critical care ultrasonography (thoracic, abdominal, and vascular) and echocardiography (basic and advanced), each with defined competences [157].

The point-of-care ultrasonography (POCUS) can be used as a fast and reliable diagnostic tool that narrows differential diagnosis in acute instable patients, and guides emergency medical procedures (drainage of cardiac tamponade, haemo- or pneumothorax, etc.) [158]. POCUS is of particular relevance in evolving shock, when the further therapy depends on the underlying pathophysiology (distributive, cardiogenic, hypovolemic and obstructive). Furthermore, transthoracic and transoesophageal echocardiography are standard diagnostic tools for acute cardiac pathologies, hemodynamic status assessment, monitoring and treatment guidance in the modern critical care [159]. Routine critical care echocardiography is being recommended for all non-cardiac and non-cardiothoracic critically ill patients to evaluate cardiac function as it can recognize regional wall abnormalities instantaneously after the onset of cardiac ischemia [160]. Such a quick recognition can fasten interventions potentially leading to reduction of mortality. Patients with echocardiographic abnormalities show a significant disadvantage in ICU survival [161].

In cardiac arrest, POCUS can be immeasurably helpful in ruling out reversible causes, such as cardiac tamponade, tension pneumothorax or pulmonary embolism, leading to adapted and immediate therapy [21]. Moreover, POCUS is used in the prognosis of cardiac arrest, as the absence of organized contractions of the heart muscle after three circles of advanced life support suggests a negligible likelihood of the return of spontaneous circulation [162,163,164,165]. This can further help in decision-making of other invasive measurements like initiation of extracorporeal membrane oxygenation.

During the ongoing health crisis, ICUs reached their capacity limits. This necessitated diagnostic methods providing the fast diagnosis of potential complications and disease progress. Especially lung US presents a possible, if not even superior alternative to computed tomography scan or pCXR for the evaluation of COVID-19 pneumonia, especially in a resource-limited settings and vulnerable groups (pregnant, children) [166,167,168,169,170,171]. POC diagnostics decrease the need for patient relocations and thereby the potential risk of transport related adverse events and further infection transmission while saving time.

Furthermore, the use of POCUS in COVID-19 patients may reduce the total amount of emitted ionising radiation and minimize the time radiographers spend on infected wards and use of personal protective equipment [172,173]. During pandemic times, the constrained resources of sectional imaging devices can be kept back for non-COVID patients, while in the meantime unused sonographic devices from less busy departments, e.g., outpatient clinics, are provided to COVID-departments [174].

In polytraumatized intensive care patients, POCUS is predominantly used for fast detection of possible causes of acute hemodynamic instability (intraperitoneal bleeding, cardiac tamponade, haemothorax or pneumothorax). This evaluation is commonly called the “extended focused assessment with sonography for trauma–eFAST” [175]. The eFAST includes six-view US examination, starting with the hepatorenal space (Morrison’s pouch), perisplenic space (Koller pouch), suprapubic (Douglas space), subcostal space, and finally views of each hemi-thorax. In case of an instable patient and positive eFAST, an emergency surgical intervention is most likely indicated even without computed tomography confirmation [176].

Finally, POCUS represents a revolutionary and safe diagnostic technique, without risk of malignity or negative effects due to exposure accumulation. Special attention should be given to the guidelines in the case of eye, lungs (due to risk of capillary haemorrhage), and fetus examinations [177].

A further important bedside diagnostic and therapeutic tool in the care of critically ill patients is a flexible bronchoscopy. In the last decade, it became a standard of care for diagnostic and interventional airway procedures. Flexible bronchoscopy is used in the case of aspiration, bleeding, strictures, lobar collapse, atelectasis and foreign body aspiration, as well as during airway assessment and management (trauma, tracheostomy, acute inhalation injury, burns, monitoring after lung transplantation, difficult airway, double lumen tube insertion, etc.) and finally for bronchial lavage, biopsy and probe sampling [178]. In the prehospital setting, disposable and portable bronchoscopy is used increasingly and may prove to be a reliable marker of intubation success [179]. However, bronchoscopy is a quite invasive procedure mostly performed on already respiratory compromised patients. Therefore, the risk of complications, mainly hypoxia must be carefully weighed against its potential benefits [180].

## 5. COVID-19 Point-of-Care Diagnostic

The SARS-CoV-2 virus, firstly registered in Wuhan (China), is a respiratory virus presenting an international threat to public health and has been declared by the WHO a pandemic in March 2020 [181]. The WHO reported over 257,000,000 confirmed cases of infections and more than five million confirmed deaths due to COVID-19 disease as of 22 November 2021 [181]. The global overload of ICU capacities and shortage in personal protective equipment led and still leads to massive cancelation of elective surgeries, to rationalization of available critical care beds and respirators [182]. Primary practice, emergency medical services and emergency departments faced rising numbers of persons in need of rapid and reliable SARS-CoV-2 virus diagnostics. Due to the shortage of kits for molecular testing, many emergency departments implemented screening with antigen tests to save time and laboratory resources [183].

As a result of the fast progressing COVID-19 pandemic and vast number of persons with suspected SARS-CoV-2 infections, laboratory capacities of the gold standard—the reverse transcription polymerase chain reaction (rt-PCR) testing– are limited. Furthermore, this is associated with long turnaround times and restricted availability due to general laboratory rt-PCR accessibility. The delay of the definite diagnosis may result in prolonged and unnecessary isolation of patients, the use of personal protective equipment and the use of already limited ICU COVID-19 determined beds. Therefore, enormous scientific and commercial effort is invested in the development of rapid and reliable POC tests that would shorten turnaround time with acceptable price, sensitivity and specificity, simple sampling, being widely available and with technology being easy to use. Many hospitals implemented SARS-CoV-2 virus screening strategies, where every person entering the hospital has to be in the possession of a negative SARS-CoV-2 test result, a certificate of recovery or vaccination. In out-patient departments, each patient in a life-threatening condition has to be tested on SARS-CoV-2. Until a result is available, personal protective equipment is strongly recommended. Therefore, every patient entering ICU should be already tested in the setting of primary evaluation. However, due to the incubation period, repeated testing is often needed, where the use of POC tests can save time, costs and make laboratory capacities more available for other tests.

**Figure 2:**
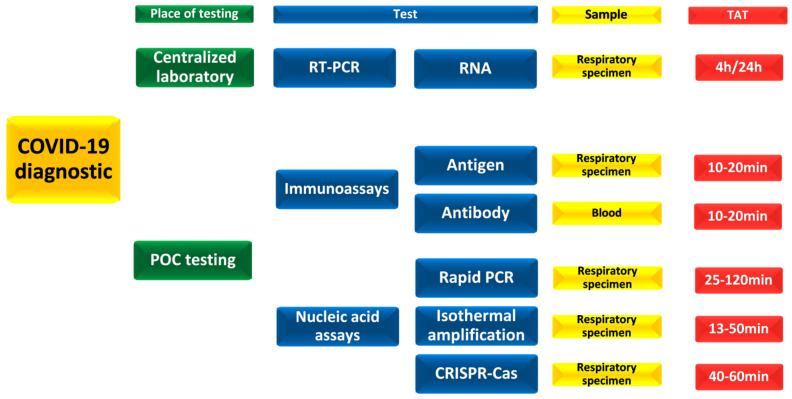
COVID-19 diagnostic possibilities, sampling and turnaround time [184,185]. POC-point of care, TAT–turnaround time, RT-PCR-reverse transcription polymerase chain reaction, RNA-ribonucleic acid, PCR-polymerase chain reaction, CRISPR-Cas-clustered regularly interspaced short palindromic repeats protein; respiratory specimen (nasopharyngeal and oropharyngeal swabs, saliva, bronchoalveolar lavage, tracheal aspirate).

The available alternatives of rt-PCR in the acute setting are POC immunoassays and nucleic acid assays, presented in Figure 2. The most often used method in critical care is nasopharyngeal or oropharyngeal sampling with antigen assays, where the specific monoclonal antibodies bind to the SARS-CoV-2 virus antigens [184]. Numerous antigen test kits can be found on the market providing results in 10 to 30 min with a 100% specificity and acceptable sensitivity of 88–94%. The main advantage of antigen assays is speed, low complexity of sampling, acceptable price and no need for special equipment [184]. As the SARS-CoV-2 pandemic progresses, commercial interest is rising, and numerous kits are available on the market resulting in wide availability and a great alternative for SARS-CoV-2 virus screening.

However, further development of reliable diagnostic methods with massive testing capacities remains the major challenge of the COVID-19 pandemic. One of the main goals of POC evolution is the development of a rapid and cheap molecular amplification-based test, offering higher sensitivity and a perfect alternative to antigen tests [186]. An important limitation may be the cost, as wide use of POC devices often presumes affordable assays [187]. Other challenges of COVID-19 diagnostics are the specimen sampling and logistics related to sample pretreatment and processing. Saliva could be seen as a possible alternative to nasopharyngeal swabs, reducing costs and increasing speed. It is an uncomplicated sampling technique with the potential for mass testing. The low sensitivity of the assay presents its main disadvantage at the moment, implying a need for further improvement. In this direction, certain molecular techniques for the detection of SARS-CoV-2 in saliva have been developed, requiring very small virus concentrations and avoiding sophisticated pretreatment of the original sample [186].

The COVID-19 pandemic overwhelmed the vast majority of healthcare systems worldwide, showing that a better readiness is needed for successful prevention, rapid diagnostic and treatment of novel disease outbreaks. Further development should focus on molecular or antigen assays for at-home testing, reducing unnecessary contacts, and possible spread of infection. The main challenge may be to ensure correct and adequate sample collection and to avoid potential harm while being widely accessible and cheap [186]. Identification of virus particles in the air may be an alternative approach with the possibility of widespread use [186]. However, the high ability of mutation of SARS-CoV-2 poses a major challenge in development of POC diagnostic devices as these assays need to discriminate different virus variants or even other viruses [186,188].

Innovative approaches for future POC diagnostic assays of emerging and new respiratory viruses can be seen in the use of biosensors, capillary convective PCR techniques, giant magnetoresistive biosensors, lateral flow assays, and other techniques systematised by Nelson et al. [189]. The technology developed for the detection of certain viruses could be adapted and transmitted into devices capable of other pathogens detection [189]. For example, the experimental use of biosensors with electrochemical assays being able to bind to viral antibodies may be expanded to multiple viruses’ detection at the same time improving efficiency and leading to cost reduction [189]. Furthermore, laboratory-based nucleic acid amplification tests (reverse transcription strand invasion-based amplification, reverse transcription loop-mediated isothermal amplification, reverse transcription-based recombinase polymerase amplification, etc.) have a potential for POC application and could be incorporated into microfluidic chips, if the technique of nucleic acids extraction is conquered [189].

The fast development of technology, machine learning, and artificial intelligence has an important impact on POC devices evolution, with new devices being more remote, user-friendly, automated and with internal control, providing rapid results in a low cost and efficient way [186]. An additional advantage of automatized analysis and remote reporting is the data handling, resulting in rapid data transmission and the potential of better prevention and pandemic control [190]. Manual reading of results, with further data processing and interpolation in official databases is a slow, expensive and demanding process. Artificial intelligence with deep learning methods may advance POC technology revolutionising medical diagnostics and reducing the workload of healthcare workers and authorities [191,192,193].

## 6. Future Development and Outlook

This review summarizes the most promising POC diagnostic approaches in critical care, specially focusing on laboratory monitoring and imaging procedures with a short outlook of COVID-19 POC diagnostic possibilities in the acute setting, outlining up to date information and literature sources on most actual standard of care and use of POC diagnostic. Moreover, we abridged possible advantages and disadvantages of POC and central laboratory approach, and discussed the usefulness of immediate diagnostic of life-threatening medical conditions using POC in the acute and critical care setting. The field of POC diagnostic is experiencing a period of rapid expansion, being driven by new evidence for clinical effectiveness, increased accuracy and speed, reduced cost and new technologies allowing consolidation of testing into even smaller devices. These technological improvements have a potential to further facilitate the transition of centralized testing to the bedside.

Besides development of new POC diagnostic possibilities, further research should focus on improvement in accuracy, performance, reduced sample volume, speed and reliability of POC devices. These devices should be robust in all ways, low-cost, not requiring special storage conditions and easy to use for people with minimum training, ideally including internal control that can exclude invalid tests.

The use of nanomaterials and microfluidics resulted in increased sensitivity of tests, holding a great promise as a future of more economical POC devices with even shorter turnaround time. A number of new highly pursued features of POC biosensors are being continuously developed and presented to the scientific community [194]. In a recently published review from Campuzano et al., the use of antibiofouling (antibiofouling polymers are being increasingly used in nanomedicine and macroscopic surface coatings, with poly(ethylene glycol) as the most widely-used polymer) [188,195,196], aptamer and biomolecular switches (molecular recognition receptors for electrochemical biosensors, with near real-time response) [188,197], next generation of amplification-free nucleic acid detection techniques (as an alternative to delicate and time-consuming rt-PCR, with immense importance in a resource-limited setting) [198,199] and other novel technologies are presented, with the main features that POC devices should comply with [188]. Further information on POC biosensors is outside of the scope of this review.

We can expect that the future development will result in expansion of test menus, shorter analysis time, ease of use and even smaller and more portable devices with automated and regular quality checks. Continuous monitoring devices, providing live-time results, are already available, but at a very high cost of use and still unclear precision and benefit, needing further research and development. Nowadays, most of the POC diagnostic results are automatically available in a patient’s electronical medical charts, showed on the patient’s monitor or even sent to a physician directly or analysed through artificial intelligence programs. The influence of artificial intelligence is already seen in automatic analysis and interpretation of medical imaging, leading to reduced turnaround time and costs, while improving accuracy [152,200].

Finally, the further development of POC in critical care should focus on bringing even more resources to the patient, which may lead to faster diagnostic and increased patient safety.

## 7. Conclusions

The use of POC devices in the care of the critically ill is strongly recommended, when the continuing education and training of healthcare workers is also provided. These diagnostics can be of enormous help in the hands of experienced intensivists, but the most advanced technology cannot be adequately employed without the needed expertise and routine. The continuing education, simulation and training should be broadly implemented and offered, encouraging healthcare workers to expand their foundations of knowledge and stay up to date on the newest developments. While the POC diagnostic may not necessarily replace centralized diagnostics, it is becoming an important and indispensable modality for improving care and outcomes of critically ill patients.

## Figures and Tables

**Table 1:** Advantages and disadvantages of point-of-care diagnostics in the intensive care setting.

Perspective	Advantages	Disadvantages
Patient		
	Fast diagnosis	Cost of POC
	Reduced treatment delay	Need for additional diagnostics
	Reduced morbidity and mortality	Quality of results and related risk
	Reduced length of stay	
	Smaller sample volume	
	Improved patient care and treatment outcomes	
	Avoiding patient and sample misidentification	
	Avoiding patient relocation	
	Patient safety	
Healthcare workers		
	Early recognition of life-threatening conditions	Limited diagnostic possibility
	Immediate and guided treatment of life-threatening conditions	Technical support not immediately accessible
	Immediately available results	Increased work load for ICU personal
	Improved staff efficiency	Storage of equipment
	Eliminated manual transcription of results	Maintenance
	Reduced turnaround time	Calibration and regular quality check
	Precise results due to immediate analysis (blood gas)	Training and recertification for POC technology
	Reduction of need to leave the patient	Results quality
	Improves efficiency of laboratory staff by reducing work load	Misinterpretation of results due to missing expertise
	Reduced administrative work	Exposition to radiation hazard
	Avoiding laboratory work process interruptions due to urgent sample analysis	Handling of biohazard waste
	Avoiding lost sample scenarios	
	Avoiding potential technical problems in steps of sample processing	
	Excluding transport and logistic issues	
	Excluding laboratory result communication from	
	portable POC devices	
	Improved general efficiency and productivity	
Government or healthcare funder		
	Reduced cost of care due to: - Reduced morbidity - Reduced length of stay - Reduced use of central laboratory - Avoiding unnecessary advanced diagnostic	Cost of POC for: - Research and development - Training and recertification - Equipment - Maintenance
	Reduced loss of productivity due to sick leave Lower costs due to faster termination of work cessation	Risk of unnecessary testing and overtesting
ICU-Intensive care unit; POC-point-of-care		

## Data Availability

Not applicable.
